# Supplementary material for: Characterization and Assembly Dynamics of the Microbiome Associated with Swine Anaerobic Lagoon Manure Treated with Biochar
Source: Microorganisms. 2025 Mar 27;13(4):758. doi: 10.3390/microorganisms13040758 (PMC12029491; doi:10.3390/microorganisms13040758)
Supplement: Supplementary file 1 [file microorganisms-13-00758-s001.zip › microorganisms-3516148-supplementary.pdf]

Table S1. Gaseous flux percent reductions (%R) and pH levels of various biochar treatments on swine manure at day 14.

**Sample**	**NH_3 Flux %R***	**CH_4 %R**	**H_2O %R**	**CO_2 %R**	**pH Top**	**pH Bottom**
CON-1	NA	NA	NA	NA	9.579	9.579
CON-2	NA	NA	NA	NA	9.48	9.48
CON-3	NA	NA	NA	NA	9.614	9.614
CON-4	NA	NA	NA	NA	9.525	9.525
COR-Biochar-1	98.94357	73.81895	29.02189	-80.4753	9.372	9.38
COR-Biochar-2	-5.40485	80.26083	11.99211	-118.189	9.293	9.276
COR-Biochar-3	50.57933	86.24378	26.76028	-65.427	9.345	9.348
COR-Biochar-4	-5.40553	75.18167	11.48288	-71.3018	9.322	9.312
FIN-Biochar-1	49.42067	86.3952	24.97569	-58.0476	9.319	9.32
FIN-Biochar-2	33.53326	86.19048	22.30906	-74.4696	9.27	9.254
FIN-Biochar-3	62.13877	95.805	11.49473	-19.739	9.349	9.34
FIN-Biochar-4	36.55262	84.45781	100	-277.045	9.259	9.244
UF-Biochar-1	67.10742	93.92112	34.6679	-92.4859	9.393	9.381
UF-Biochar-2	65.49209	93.51327	-50.2111	-52.8973	9.371	9.311
UF-Biochar-3	38.39967	97.29482	5.591564	-74.013	9.356	9.343
UF-Biochar-4	-27.4741	88.11421	9.127814	-97.5686	9.307	9.31

*%R calculated from equation 1.

Table S2. Dunn's test for comparisons between treatments and flux percent reduction (%R) for CH4, H2O, and CO2.

**Measurement**	**Comparison**	**Z**	***p*-value**	**FDR adjusted *p*-value**
Methane (CH_4)	COR-Biochar – CON	1.3466	0.1781	0.2671
	COR-Biochar – FIN-Biochar	-1.1221	0.2618	0.3141
	CON – FIN-Biochar	-2.4688	0.0136	0.0401
	COR-Biochar – UF-Biochar	-2.0199	0.0434	0.0868
	CON-Biochar – UF-Biochar	-3.3666	0.0007	0.0046
	FIN-Biochar – UF-Biochar	-0.8978	0.3693	0.3693
Ammonia (NH_3)	COR-Biochar – CON	0.8229	0.4105	0.8211
	COR-Biochar – FIN-Biochar	-0.5985	0.5495	0.8243
	CON – FIN-Biochar	-1.4215	0.1552	0.9311
	COR-Biochar – UF-Biochar	-0.5237	0.6004	0.7206
	CON-Biochar – UF-Biochar	-1.3466	0.1781	0.5343
	FIN-Biochar – UF-Biochar	0.0748	0.9404	0.9404
Water (H_2O)	COR-Biochar – CON	2.3192	0.024	0.0611
	COR-Biochar – FIN-Biochar	-0.2244	0.8224	0.8224
	CON – FIN-Biochar	-2.5436	0.0109	0.0658
	COR-Biochar – UF-Biochar	1.1970	0.2313	0.3469
	CON-Biochar – UF-Biochar	-1.1222	0.2618	0.3141
	FIN-Biochar – UF-Biochar	1.4215	0.1552	0.31037
Carbon Dioxide (CO_2)	COR-Biochar – CON	-2.5436	0.0109	0.0658
	COR-Biochar – FIN-Biochar	-0.3741	0.7084	1
	CON – FIN-Biochar	2.1696	0.03	0.06
	COR-Biochar – UF-Biochar	-0.0748	0.9404	0.9404
	CON-Biochar – UF-Biochar	2.4688	0.0136	0.0407
	FIN-Biochar – UF-Biochar	0.2993	0.7647	0.9177

Table S3. NST metrics for assessing stochasticity in microbial assembly.

Treatment	NSTi	MST	SES	β_{RC}
AMB	0.809	67.3%	0.5634	0.2883
CON	0.7284	64.2%	0.4985	0.2483
COR-Biochar	0.7634	72.7%	0.5148	0.2483
FIN-Biochar	0.8425	71.7%	0.5447	0.255
UF-Biochar	0.8576	72.6%	0.559	0.2683

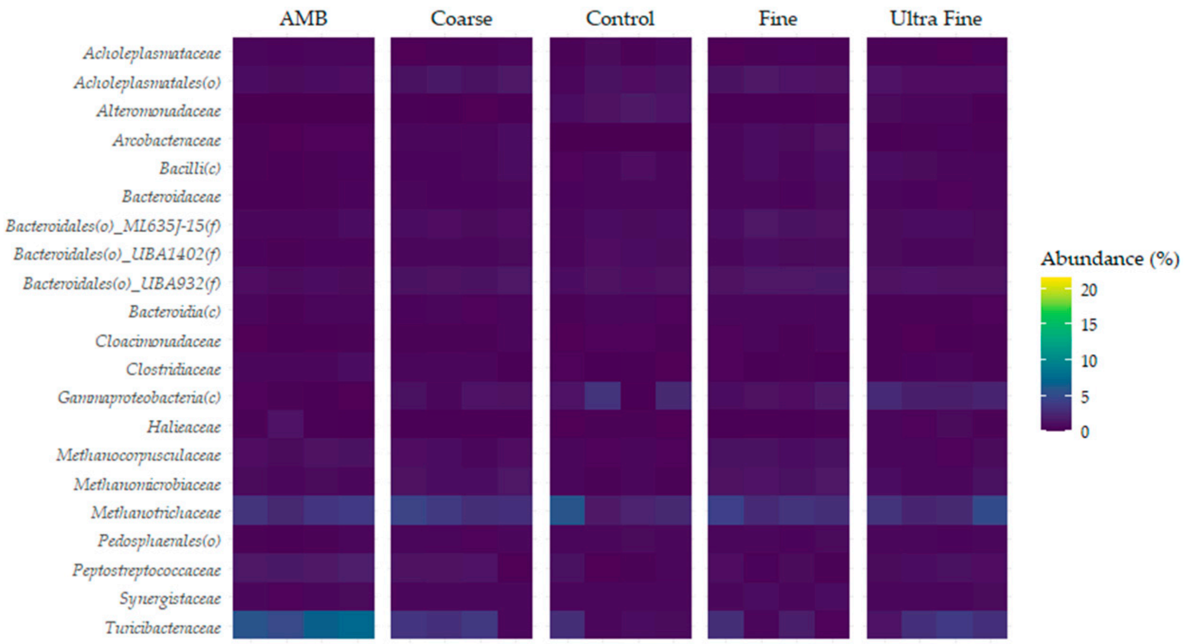

Figure S1. Family level taxonomic profiles for each treatment type.

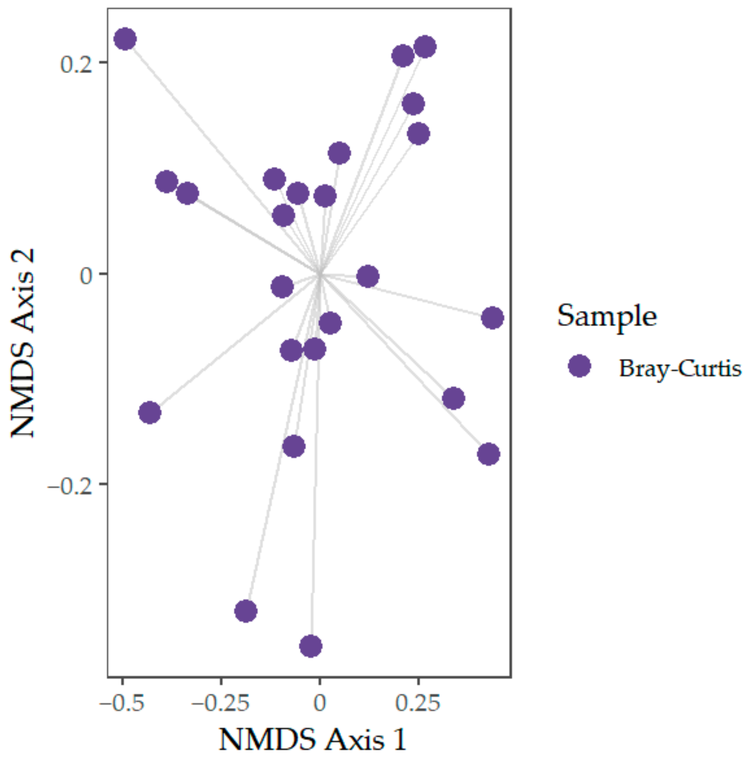

Figure S2. NMDS plot for βRC values using the Bray-Curtis distance matrix.

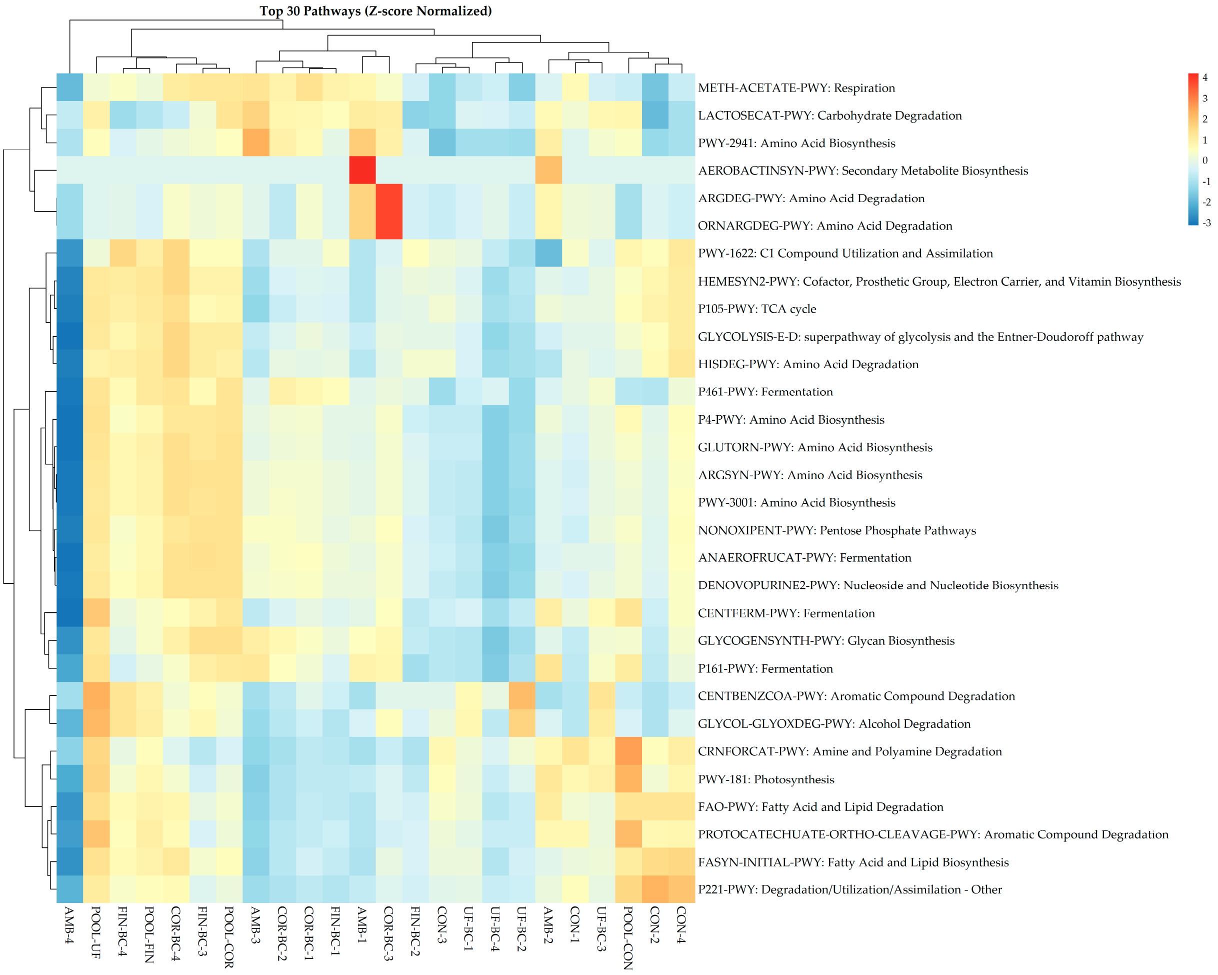

Figure S3. The top 30 predicted pathways using PICRUSt after Z-score normalization.
